# Phytosterols in Seaweeds: An Overview on Biosynthesis to Biomedical Applications

**DOI:** 10.3390/ijms222312691

**Published:** 2021-11-24

**Authors:** Soo-In Sohn, Periyasamy Rathinapriya, Sekaran Balaji, Devasahayam Jaya Balan, Thirukannamangai Krishnan Swetha, Ravindran Durgadevi, Selvaraj Alagulakshmi, Patchiappan Singaraj, Subramani Pandian

**Affiliations:** 1 Department of Agricultural Biotechnology, National Institute of Agricultural Sciences, Rural Development Administration, Jeonju 54874, Korea; 2 Department of Biotechnology, Alagappa University, Karaikudi 630 003, India; rathina.priya25@gmail.com (P.R.); balanjaya38@gmail.com (D.J.B.); swethakrishnan02@gmail.com (T.K.S.); devdurga636@gmail.com (R.D.); lakshmivinay.317@gmail.com (S.A.); 3 Department of Biotechnology, Vidhyaa Giri College of Arts and Science, Karaikudi 630 003, India; 4 Independent Researcher, Madurai 625 020, India; balajisekar91@yahoo.in (S.B.); singam.biotech@gmail.com (P.S.)

**Keywords:** antioxidants, antimicrobials, β-sitosterol, functional foods, phytosterols, seaweeds

## Abstract

Seaweed extracts are considered effective therapeutic alternatives to synthetic anticancer, antioxidant, and antimicrobial agents, owing to their availability, low cost, greater efficacy, eco-friendliness, and non-toxic nature. Since the bioactive constituents of seaweed, in particular, phytosterols, possess plenty of medicinal benefits over other conventional pharmaceutical agents, they have been extensively evaluated for many years. Fortunately, recent advances in phytosterol-based research have begun to unravel the evidence concerning these important processes and to endow the field with the understanding and identification of the potential contributions of seaweed-steroidal molecules that can be used as chemotherapeutic drugs. Despite the myriad of research interests in phytosterols, there is an immense need to fill the void with an up-to-date literature survey elucidating their biosynthesis, pharmacological effects, and other biomedical applications. Hence, in the present review, we summarize studies dealing with several types of seaweed to provide a comprehensive overview of the structural determination of several phytosterol molecules, their properties, biosynthetic pathways, and mechanisms of action, along with their health benefits, which could significantly contribute to the development of novel drugs and functional foods.

## 1. Introduction

Marine macroalgae (or seaweed) hold immense nutritional value and have constituted an important position in Asian diets since time immemorial. Traditionally, seaweeds *viz.*, *Undaria pinnatifida* (wakame), *Cladosiphono kamuranus* (mozuku), *Laminaria japonica* (kombu) and *Gelidium crinale* (tengusa) are consumed as healthy foods in Japan [1]. *Sargassum fusiforme*, in addition to being one of the edible seaweeds consumed in Korea and China, holds an important position in traditional Chinese medicine due to its anti-atherosclerotic activity [2]. In recent times, the demands for seaweed in other parts of the world, such as North and South America, have also increased due to the migration of traditional seaweed consumers. Moreover, France has recently approved the human consumption of seaweed as condiments and vegetables, which further increases the value of seaweed in the global food market. Although seaweed has been consumed since prehistoric times, its commercial utilization in food, cosmetics, and pharmaceutical industries was recognized later [3]. Seaweed extracts are generally rich in natural growth hormones, nutrients, and trace minerals. Among several other compounds, the nutritional value of seaweed is mainly attributed to the presence of phytosterols. Phytosterols, as the name implies, are defined as fatty compounds produced by plants, and remarkably contribute as the major lipid constituent of biological membrane of plant cells. Although these plant steroids have similar chemical structures like cholesterol, the differentiation in C_24_ side chains makes them metabolically and functionally distinct from each other [4]. Like terrestrial plants, seaweeds as well, exhibit the diversified composition of phytosterol contents with a similar profile of health benefits and their prevalence in this phytoplankton is largely influenced by their evolutionary origin [5,6]. For instance, brown algae (phaeophyta) predominantly contain phytosterols such as fucosterol and brassicasterol, with a small proportion of plant cholesterol and therefore considered a promising source for phytosterols. In contrast, red algae (rhodophyta) contain cholesterol as their principal sterol content, with a minor quantity of phytosterols such as sitosterol, fucosterol, chalinasterol and desmosterol. On the other hand, green algae (chlorophyta) vary in their types of sterols, such as ergosterol, chondrillasterol, β-sitosterol, 28-isofucosterol, cholesterol and poriferasterol, depending on the species [7,8]. Furthermore, to date, there have been no studies found to demonstrate the obvious negative effects (in terms of toxicity) of phytosterols on humans. Accordingly, international agencies such as the Food and Drug Administration (FDA) and the European Union Scientific Committee (EUSC) have already approved phytosterols as safe to use [9]. Despite the myriad of research interest in phytosterols, there has been a lacuna in an up-to-date literature survey elucidating their biosynthesis, pharmacological effects and other biomedical applications. Therefore, the goal of this review is to provide an overview of the biosynthesis, extraction, and characterization of phytosterols in seaweed, as well as of their biological properties with biomedical applications, for effective seaweed utilization in the food and pharmaceutical industries.

## 2. Biosynthesis of Phytosterols

The sterol biosynthesis pathway is an important pathway of living organisms, exhibited by certain bacteria and all eukaryotes [10,11]. Recent discoveries have increased the complexity of this pathway, urging researchers to decipher it further in order to deepen perceptions of its activity. Phytosterol biosynthesis is a branch of sterol synthesis found in almost all plant species and it can be distinguished from the sterol biosynthesis pathway of all eukaryotic kingdoms, such as animals and fungi. Although seaweeds are known to be potential producers of phytosterols, not all species of seaweeds have evolved to do so. Only limited species, such as brown seaweed, have the ability to produce phytosterols such as fucosterol and saringosterol. These sterols have therapeutic implications, such as neurostimulatory effects, and thus have piqued the interest of the clinical community [12,13]. The paucity of investigation and the diversity of macroalgal species have obscured the identification of pathways responsible for phytosterol synthesis. Nevertheless, Calegario et al. [14] postulated that seaweeds may use the traditional pathways of plants for isoprene unit synthesis. As in plant species, the phytosterol biosynthesis in macroalgae can be stratified into three major segments: (i) the biosynthesis of isoprene, (ii) the condensation of isoprene into triterpenoids and their epoxidation and (iii) the biosynthesis of phytosterols [15]. In these sequential reactions, products of glycolysis are converted into hydrocarbons such as isoprene to triterpenoids and final end products of phytosterols [16].

### 2.1. Biosynthesis of Isoprene

Isoprene is the basic unit of all isoprenoids and terpenoids with a five-carbon functional group [17]. In a eukaryotic cell, the synthesis of isoprenes, namely isopentenyl diphosphate (IPP) and dimethylallyl diphosphate (DMAPP) can be carried out through two unique pathways called mevalonate (MVA) and methylerythritol phosphate (MEP) pathways. All plant species can utilize both these pathways to generate the precursors of isoprenes. In contrast, green macroalgae are found to use the MEP pathway, as they lack the genes involved in the MVA pathway. However, red algae have the potency to synthesize IPP and DMAPP through both these cytosolic- and plastid-relying mechanisms. It is postulated that during endosymbiosis, green algae could have lost machinery from the MVA pathway and evolved with most relying on the MEP pathway for all fundamental aspects of isoprenoid synthesis. Conversely, primary endosymbionts, such as plants and red algae, have retained both MVA and MEP pathways over their evolutions [18]. Most portions of the MVA and MEP pathways transpire in the cytoplasm and plastid, respectively. Interestingly, all the sequential events of the MEP pathway ensue in plastids; however, the genes involved in these processes are encoded by the nuclear genome [19,20]. Furthermore, the MEP pathway produces both IPP and DMAPP directly, whereas MVA produces only IPP that is followed by isomerization of IPP into DMAPP by IPP isomerase (IPPI). As green algae rely solely on the MEP pathway for isoprenoid synthesis, they have evolved to express chloroplast membranal antiporters to maintain a balanced flux of IPP/DMAPP between plastid and cytosol [21]. Though the MEP pathway produces both these precursors, the ratio of IPP: DMAPP generated through this event varies among plant species.

**Figure 1:**
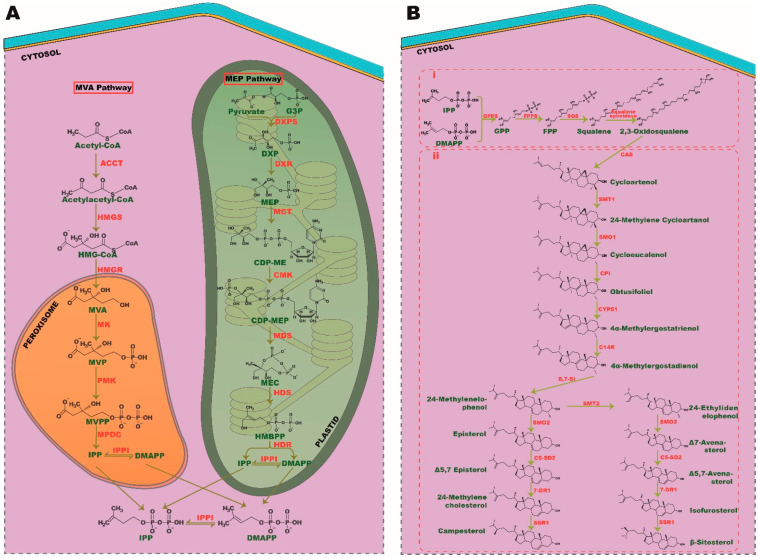
Biosynthesis pathways of phytosterols. (**A**) Isoprene unit, IPP, and DMAPP synthesis. A product of glucose metabolism, acetyl-coA and pyruvate is converted into isoprene units through MVA and MEP pathway, respectively. (**B**) (i) Condensation of isoprene into triterpenoids and (ii) phytosterol synthesis. SMT, sterol C-24 methyl transferase; SMO, sterol C-4 methyl oxidase; CPI, cyclopropyl sterol isomerase; CYP51, sterol C-14 demethylase; C-14R, sterol C-14 reductase; 8, 7-SI, sterol 8, 7 isomerase; C5-SD2, sterol C5(6) desaturase; 7DR1, 7-dehydrocholesterol, SSR1, sterol side-chain reductase 1.

The MVA pathway comprises six enzymatic reactions, which commence with acetyl-CoA condensation by acetyl-coA C-acetyltransferase (ACCT), followed by synthesis of 3-hydroxy-3-methylglutaryl-CoA (HMG-CoA) by HMG-CoA synthase (HMGS) with subsequent reduction by HMG reductase (HMGR) to form MVA as an intermediate. MVA is then pyrophosphorylated and decarboxylated in subsequent steps by mevalonic acid (MK), phosphomevalonate (PMK) kinases and mevalonate-5-diphosphate decarboxylase (MPDC), respectively, to form the final isoprene unit, IPP. In the MEP pathway, the final product pyruvate, and intermediate- glyceraldehyde-3-phosphate (G3P), of glycolysis are converted into isoprene via seven successive steps. Pyruvate and G3P are condensed together to produce 1-deoxy-D-xylulose 5-phosphate (DXP) by DXP synthase (DXS), followed by reduction, cytidylation, phosphorylation, decytidylation and final reduction by DXP-reductoisomerase (DXR), 2-C-methyl-derythritol 4-phosphate cytidylyltransferase (MCT), 4-(cytidine 5′-diphospho)-2-C-methyl-D-erythritol kinase (CMK), 2-C-methyl- D-erythritol 2,4-cyclodiphosphate synthase (MDS), 4-hydroxy-3-methylbut-2-enyldiphosphate (HMBPP) synthase (HDS) and HMBPP reductase (HDR), respectively, to generate the end isoprene products IPP and DMAPP (Figure 1A).

### 2.2. Condensation of Isoprene into Triterpenoids and Their Epoxidation

Isoprene, with a five-carbon atom, condenses together to generate monoterpenes (C10), sesquiterpenes (C15), diterpenes (C20), and triterpenes (C30). Triterpenes serve as precursors for phytosterol synthesis. Isoprene from the MEP pathway catalyzes the synthesis of monoterpenes, diterpenes, chlorophyll, carotenoids, and other phytohormones, such as gibberellin, strigolactone, and abscisic acid. It is indeed a case that the MVA pathway-derived isoprene is responsible for the synthesis of the phytosterol precursor, squalene (a triterpene), via geranyl pyrophosphate (GPP) and farnesyl pyrophosphate (FPP), which are the precursors of monoterpenes and triterpenes, respectively [22,23]. FPP synthase (FPPS) and squalene synthase (SQS) catalyze these sequence reactions, which follow the epoxidation of squalene into 2, 3-oxidosqualene by squalene epoxidase (Figure 1Bi).

### 2.3. Phytosterol Synthesis

The biosynthesis of phytosterol exhibits a parallel pathway of cholesterol synthesis in eukaryotic cells [24]; 2, 3-oxidosqualene serves as a common precursor for all sterol synthesis i.e., sterols like cholesterol in humans, ergosterol in fungi and phytosterols in plants. The biosynthesis of cholesterol and ergosterol is catalyzed by lanosterol synthase (LAS), whereas phytosterol synthesis from oxidosqualene is catalyzed by cycloartenol synthase (CAS) [25]. As in plant species, seaweeds metabolize 2, 3-oxidosqualene to phytosterol by CAS and exhibit the hybrid cholesterol pathway as well, indicating an evolutionary relationship between these two phototrophic organisms [14].

Numerous studies have highlighted the importance of the CAS gene in phytosterol biosynthesis. In extensive research by feeding experiments using [6-^13^C^2^H_3_] MVL, Ohyama et al. [26] demonstrated that a mere 1.5% of sitosterol, encoded by the LAS1 gene, emphasizes its crucial role in the biosynthesis of phytosterols. Cycloartenol is metabolized into 24-methylenelophenol through six enzymatic reactions, such as methylation, double demethylation, double isomerization, and a reduction. From 24-methylenelophenol, the pathway branches off into two separate pathways, giving rise to episterol and Δ7 avenasterol via 24-ethylidenelophenol and end up in campesterol and β-sitosterol, respectively. Stigmasterol is synthesized directly from -sitosterol, whereas campesterol is converted into brassinolide through subsequent reactions [24] (Figure 1Bii).

## 3. Extraction and Characterization of Phytosterols

**Figure 2:**
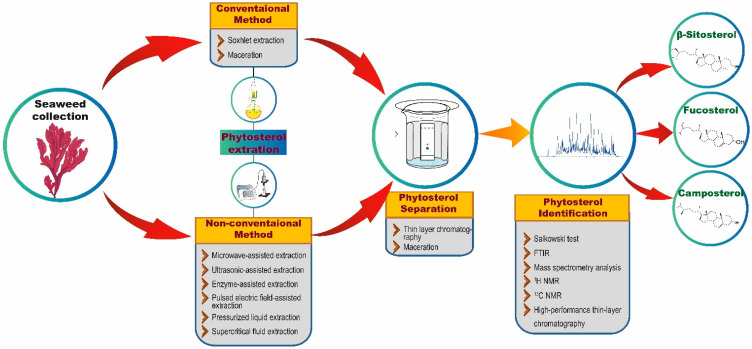
An overview of phytosterol extraction and characterization methods.

Extraction techniques are proficient in separating the soluble metabolites of seaweed using suitable solvents [27]. The isolation techniques to be used for the extraction of phytosterols generally depend on the type of phytosterols (free, glycosylated, and esterified) and the nature of the matrix [28]. In each technique, optimized experimental conditions and adequate parameters are required to attain the appropriate quantity and higher yields from seaweed extract. The quality of an extract is substantially influenced by various factors, such as plant/seaweed material, solvent, extraction procedure, and others [29]. There are two common extraction techniques, namely conventional and non-conventional extraction (Figure 2) [30]. Conventional techniques usually employ a large volume of organic solvents to extract adequate analytes from samples that are required for further analysis. Nonetheless, the utilization of a higher volume of organic solvents may have a negative impact on human health and the environment. Furthermore, conventional techniques are reported to possess several limitations, such as the necessity of solvents with very high purity, an extended period of time for extraction, low selectivity of extraction, requirement of solvent evaporation and thermal decomposition of heat liable compounds. These limitations demanded the discovery of new extraction techniques with relatively very low utilization of organic solvents and more advantages than conventional techniques [31].

Soxhlet extraction is one of the conventional techniques, and remains the most preferred extraction technique for phytosterols, still, today [32,33]; it serves as a standard to many newly developed extraction techniques (Table 1) [31]. For example, Poulose et al. [32] extracted phytosterols from the red seaweed *Gelidium spinosum* using the Soxhlet method and revealed the presence of stigmasterol, with a mass of 412.69 g/mol, through Fourier transform infrared (FTIR) and gas chromatography–mass spectrometry (GC–MS). In addition, maceration is another common conventional technique, which is stated to be simple and cost-effective for phytosterol extraction [34].

Apart from these conventional techniques, phytosterol extraction has also been shown to be accomplished using many other non-conventional extraction techniques, such as microwave-assisted extraction (MAE), ultrasonic-assisted extraction (UAE), enzyme-assisted extraction (EAE), pulsed electric field-assisted extraction (PEFAE), pressurized liquid extraction (PLE), and supercritical fluid extraction (SFE) (Figure 2) [31]. For instance, Xiao et al. [52] have employed MAE with high-speed countercurrent chromatography (liquid-liquid partition chromatography) and a UV detector to extract, separate, and purify phytosterols from the edible brown seaweeds *S. fusiforme* and *U. pinnatifida.* By employing these extraction and chromatographic techniques, the authors were able to obtain 1.5 mg of 24-methylenecholesterol and 13 mg of fucosterol from 15 g of *U. pinnatifida* and 0.3 mg of 24-methylenecholesterol and 4.6 mg of fucosterol from 15 g of *S. fusiforme*. Remarkably, Roiaini et al. [53] have performed a comparative analysis of various phytosterol extraction techniques on cocoa butter such as Soxhlet, ultrasonic, supercritical carbon dioxide, and supercritical carbon dioxide with co-solvents. The authors concluded that the highest phytosterol content was obtained when using supercritical carbon dioxide with a cosolvent.

The separation process usually follows the extraction process. In this context, thin-layer chromatography (TLC) and high-performance liquid chromatography (HPLC) techniques are extensively used separation techniques (Figure 2). After the separation process, phytosterols in the analytes are confirmed via various analytical methods. Preliminary screening of phytosterols can be performed through Salkowski test [54], in which the formation of brown ring confirms the presence of phytosterols. Besides the conventional Salkowski method, FTIR and nuclear magnetic resonance are widely utilized to analyze phytosterols. However, precise determination of phytosterols could be achieved through employing mass spectrometry analysis [32,33]. For example, GC–MS analysis of the ethanolic extracts of 18 seaweeds revealed the presence of 14 compounds including 3 sterols, cholestanol, β-sitosterol, and fucosterol [55]. In another study, the presence of a significant amount of fucosterol in brown seaweed *C. barbata* and the highest level of β-sitosterol in *H. tuna* and *C. bursa* were revealed through GC–MS analysis [43]. Similarly, Santi et al. [33] have isolated sterols from four red seaweeds namely *P. decipiens*, *P. endiviifolia*, *I. cordata*, and *P. cartilagineum* using alkaline hydrolysis extraction method and showed the presence of sterols such as fucosterol, β-sitosterol, stigmasterol, brassicasterol, cholesterol and campesterol through GC–MS analysis. Correspondingly, Kendel et al. [56] have also employed GC–MS analysis to reveal the presence of phytosterols such as fucosterol, isofucosterol, brassicasterol, chondrillasterol and cholest-4-en-3-one in the chloroform/methanol extracts of *U. armoricana* and *S. chordalis*.

In another study, Bouzidi et al. [57] used ^1^H nuclear magnetic resonance (NMR) to analyze fractions of the marine brown seaweed *C. foeniculacea* that were extracted using a chloroform methanol water mixture and isolated using reversed-phase HPLC (RP-HPLC). Through ^1^H NMR analysis, the authors revealed the presence of fucosterol and an epimeric combination of saringosterol in the seaweed fractions. Recently, a one-step preparative method for separation of phytosterols was put forward by Xia et al. [41] wherein the authors successfully utilized the high-speed countercurrent chromatographic method to extract fucosterol (23.7 mg), and saringosterol (3.1 mg) from the crude extract of *S. horneri* using two-phase solvent system. Furthermore, the authors have characterized the phytosterols through ^1^H and ^13^C NMR structural analysis. In addition, high-performance thin-layer chromatography (HPTLC, a technique for target-directed identification of active leads in a group of compounds) combined with biochemical and microchemical derivatizations revealed the presence of phytosterols and phenolic lipids in the ethyl acetate extract of 19 marine algae samples in addition to displaying bioactivity such as antioxidant, α-amylase and acetylcholinesterase inhibitory activities [41]. In a study by Oh et al. [47], methanolic extract of *E. stolonifera* was fractionated using various solvents and the fraction with strong bioactivity was further purified to yield fucosterol (99% purity, determined by HPLC) and identified by ^1^H and ^13^C NMR methods to study the neuroprotective effects of fucosterols.

Through various techniques, the sterol profiling of diverse seaweeds from different regions has already been done by many research groups, which has subsequently helped to gratify the demand for phytosterols in the global market. By uncovering the concentration of diverse sterols in different seaweeds of different regions during various seasons, the optimal techniques and suitable seaweeds could be easily identified for the preparation of particular phytosterols in large quantities. Furthermore, the extraction, isolation and analytical techniques are continually evolving, which simplifies the overall preparation process of phytosterols eventually and also proves to be more competent, fast and eco-friendly than conventional techniques.

## 4. Health Benefits and Biomedical Applications of Phytosterols

**Figure 3:**
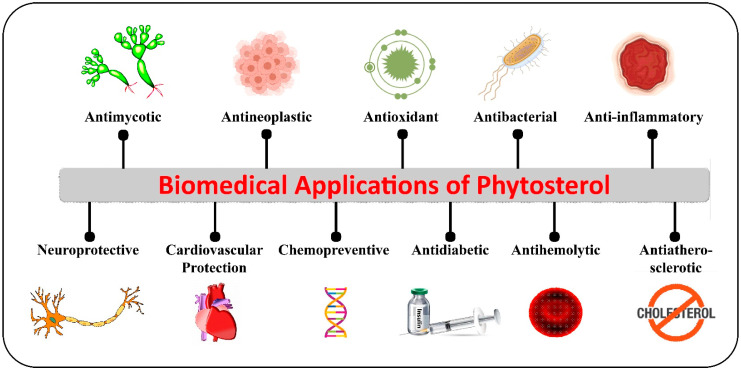
Biological properties and biomedical applications of phytosterols.

Phytosterols are renowned for their cholesterol-lowering activity, which potentially reduces the intestinal absorption of cholesterol by up to 30–50%. One of the major mechanisms driving the hypocholesterolemic activity of phycosterols is reported to be related to the competition of phytosterols with structurally similar mammalian cholesterol in the intestinal lumen, which, in turn, reduces the quantity of cholesterol accessible for intestinal absorption. Several other mechanisms underlying the hypocholesterolemic activity of phytosterols have also been previously reported, which include (i) alterations in the expression level of genes encoding sterol-carrying proteins, such as the ATP-binding cassette transporters ABCG5 and 8, that increase the discharge of cholesterol from enterocytes into the intestinal lumen or Niemann–Pick C1-like 1 protein that decrease the transfer of cholesterol to enterocyte; (ii) a reduction in the esterification of cholesterol in enterocyte; and (iii) an increase in the level of cholesterol elimination from the body via transintestinal cholesterol excretion [58]. For instance, the intragastric administration of sitosterol/ fucosterol (25 mg) and cholesterol (25 mg) encompassing an emulsified lipid meal was identified to interfere with the absorption and micellar solubility of cholesterol in rats [59]. Additionally, phytosterols are also reported to act as anticonvulsants, antidepressants, hepatoprotectants, angiotensin-converting enzyme inhibitors, and regulators of cholesterol homeostasis [14]. For example, the *S. fusiforme*-derived fucosterol was found to exhibit antidepressant activities in mice by curbing the immobility time for 30 min in tail suspension test and forced swimming test. The pathogenesis of depression is mainly hypothesized to be related to the reduction in the levels of monoamine neurotransmitters such as dopamine, serotonin and noradrenaline. Interestingly, fucosterol was found to elevate the levels of these monoamine neurotransmitters, which affirmed its antidepressant activity [60]. In another investigation, oral administration of 30 mg/kg of fucosterol from *Pelvetia siliquosa* was found to exhibit inhibitory activity against streptozotocin-triggered diabetes in rats by significantly reducing serum glucose level and the accumulation of sorbitol in lenses. Further, oral administration of 300 mg/kg of fucosterol was identified as reducing blood glucose concentration and glycogen degradation in epinephrine-induced diabetic rats [61]. Altogether, the myriad health-promoting effects of phytosterols open the door for the development of new drugs for treating several chronic diseases and the fabrication of phytosterols-enriched functional foods that could be used as a part of a healthy routine. Nevertheless, the health benefits of phytosterols are not only limited to cholesterol-lowering potential, but also extend to anti-cancer, antioxidant, anti-inflammatory, anti-adipogenic, anti-obesity, anti-diabetic, anti-Alzheimer, and anti-atherosclerosis activities. The different biological properties and biomedical applications of phytosterols are provided in Figure 3.

### 4.1. Phytosterols as Antioxidative Agents

**Figure 4:**
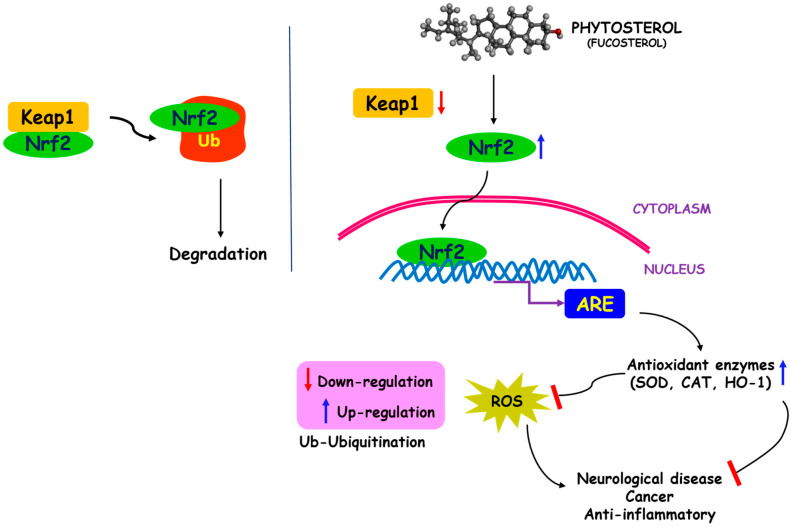
Schematic diagram representing the antioxidant mechanism of fucosterol. Up-regulation of antioxidant enzymes such as HO-1 (heme oxygenase-1), SOD, and CAT, via the Nrf2/ARE signaling pathways. The left image indicates the normal physiological conditions in which Nrf2 bound to keap1 in the cytosol undergoes degradation by the ubiquitination process.

Reactive oxygen species (ROS) and free radicals are perpetually generated and their implications in pathobiology are associated with various diseases, including cancer, neurodegenerative and cardiac diseases [62]. The human body is endogenously equipped with enzymatic antioxidants, such as superoxide dismutase (SOD), glutathione peroxidase (GPx), glutathione reductase (GR), and catalase (CAT), to counteract the effects of oxidation. A transcriptional regulator, nuclear erythroid 2-related factor 2 (Nrf2), mediates the expression of antioxidant response elements during oxidative stress [63]. In addition, non-enzymatic antioxidants are shown to boost the antioxidant defense mechanisms and thereby aid in ROS elimination. The antioxidant supplements from seaweeds are reported to reduce the risk of cancer, cardiovascular, and other degenerative diseases in humans [63,64,65]. Particularly, fucosterols from brown seaweed are gaining interest due to their exceptional antioxidant properties. Studies have suggested several mechanisms for the antioxidant activity of fucosterol, including increased activities of free radical scavenging enzymes such as GPx, CAT and SOD [61], inhibition of ROS generation by suppressing iNOS (Inducible nitric oxide synthase) and COX-2 (cyclooxygenase-2) [66,67], and inhibitory activities against biomarkers (β-secretase, acetylcholinesterase (AChE) and butyrylcholinesterase (BChE)) of Alzheimer’s disease [68]. The general antioxidant mechanism of action of phytosterol is illustrated in Figure 4.

Phaeophyta, a brown seaweed, with higher amounts of fucosterol, exhibited considerable antioxidant activity in the 2, 2-diphenyl-1-picrylhydrazyl (DPPH) assay [48]. Fucosterol, one of the major components of the brown seaweed *Padina gymnospora* ethyl acetate extract, exhibited promising antioxidant activity in β-carotene bleaching (BCB) and total reducing activity (TRA) assays [69]. Fernando et al. [70] have reported that fucosterol (from brown seaweed *S. binderi*) treatment in HaCaT keratinocytes and HDF fibroblasts cells significantly decreased intracellular ROS levels and increased cell viability during exposure to particulate matter (PM). In another study by Fernando et al. [71], fucosterol increased the level of enzymatic antioxidants, such as SOD, CAT, and HO-1, as well as the transcriptional regulator Nrf2 in A549 cells in the nucleus. Similarly, Choi et al. [72] reported that fucosterol, obtained from *E. stolonifera* and *Eisenia bicyclis*, hugely inhibited the generation of intracellular ROS and ameliorated the levels of glutathione in tert-butyl hydroperoxide and tacrine-treated HepG2 cells. Jiang et al. [73] reported the dose-dependent intensification of intracellular ROS in a fucosterol-treated HeLa cervical cancer cell line. Fucosterol from *S. horneri* showed strong free radical scavenging activities in DPPH and 2,2 azino-bis3-ethylbenthiazoline-6-sulfonic acid (ABTS) assays. In addition, fucosterol attenuated the oxidative actions in HT22 and BV2 cells by enhancing the HO-1 and Nrf2 levels in the cytoplasm and nucleus, respectively [74]. Similarly, fucosterol from *P. boryana*, a brown seaweed, significantly upregulated the antioxidant proteins such as Nrf2, and HO-1 and downregulated the Kelch-like ECH-associated protein-1 (Keap1) in the RAW 264.7 macrophage cell line induced with PM [67], in addition to anti-inflammatory activity. Remarkably, Ibrahim et al. [75] revealed the antioxidant property of sterol hydrocarbon, obtained from brown seaweed *Taonia atomaria*, through DPPH, ABTS, and total antioxidant capacity (TAC) assays. The study also unveiled the anti-inflammatory and anticancer (against human liver, breast, ovarian, and colon cancer cell lines) activities of *T. atomaria* sterol hydrocarbons. Nevertheless, it was suggested that the anti-inflammatory and anticancer activities may be due to strong antioxidant properties, indicating the effectiveness of sterols in oxidative stress-related diseases. These research findings demonstrate the overwhelming antioxidant property of phytosterols, especially fucosterol and, moreover, these antioxidants could function against oxidative stress-associated diseases. Vanbrabant et al. [76] found that 24(R, S)-saringosterol, an oxidative product of fucosterol, has higher antioxidant activity than fucosterol; however, more research is needed to confirm the antioxidant potential of 24(R, S)-saringosterol.

### 4.2. Phytosterols as Antimicrobial Agents

Phytosterols have antimicrobial activities against a wide range of human pathogens, including bacteria, fungi, viruses, and protozoa. The lipophilic extract of *Bifurcaria bifurcata,* with a considerable amount of fucosterol displayed antibacterial activities against both Gram-positive (*Staphylococcus aureus* and *Staphylococcus epidermidis*) and Gram-negative bacteria (*Escherichia coli* and *Pseudomonas aeruginosa*). Subsequently, the synergistic analysis of the extract with antibiotics significantly reduced the minimal inhibitory concentrations of the tested antibiotics [77]. Intriguingly, a cholesterol derivative, 24-propylidene cholest-5-en-3β-ol, obtained from the red algae *Laurencia papillosa* displayed antibacterial activity against *S. aureus*, *Bacillus subtilis*, *E. coli*, and *P. aeruginosa*. Furthermore, it exhibited strong antibacterial activity against the clinical isolates of *Klebsiella pneumoniae*, *Shigella flexineri*, *E. coli*, and *P. aeruginosa* [78]. In another study, assessment of the antimicrobial potential of seaweed extracts (*A. utricularis*, *C. racovitzae*, and *U. intestinalis*) against *S. aureus*, *E. coli*, and *Salmonella typhimurium* revealed noteworthy effects. Moreover, the study also revealed the promising antioxidant property of the seaweed extracts obtained from *D. confervoides* [38]. Correspondingly, *C. cylindracea* (green seaweed) extract containing sterols in major proportions has been reported to showcase antibacterial and antioxidative properties [79]. Apart from that, the evaluation of critical sterols such as campesterol, β-sitosterol, stigmasterol, epicoprostanol, etc. against a wide range of bacteria revealed notable antibacterial properties [80]. The authors also reported a higher rate of growth inhibition in Gram-negative bacteria (*Vibrio*, *Pseudomonas*, and *Bacillus* spp.) than Gram-positive bacteria. Conversely, the crude methanolic extracts of eight brown seaweeds (*Sargassum* spp., *Turbinaria* spp., and *Hormophysa cuneiformis*) had more promising antibacterial activity against Gram-positive bacteria (*S. pneumoniae, B. cereus* and *S. aureus*) than Gram-negative bacteria. Additionally, the crude extracts displayed antifungal activity against *Trichosporon mucoides*, *Candida membranaefaciens*, and *Cryptococcus neoformans*. An exploration of chemical constituents revealed fucosterol as the abundant compound, along with 24-ketocholesterol, saringosterol, stigmasta-5,28-dien3b-ol, and (22E)-3b-hydroxycholesta-5,22-dien-24-one [81]. Fucosterol, identified as one of the 10 constituents in methanolic extract of brown and green seaweeds—namely *P. pavonia*, and *U. lactuca*, respectively—exhibited antibacterial (against *S. aureus* and methicillin-resistant *S. aureus*), antifungal (against *C. krusie*, *C. neoformans*), and mild antimalarial (against *Plasmodium falciparum*) activities [82]. Intriguingly, the examination of the antitubercular activity of saringosterol and its epimers (obtained from *Lessonia nigrescens*) against *Mycobacterium tuberculosis* revealed MIC values of 0.25, 1.0, and 0.125 μg/mL [83].

Assessment of fucosterol (extracted from brown seaweed *Himanthalia elongate*) against Herpes Simplex Virus Type 1 (HSV-1) revealed noteworthy antiviral effects [84]. On the other side, fucosterol from *Fucus vesiculosus* (1.0% concentration) inhibited 100% of macroconidia growth and exhibited strong antifungal properties against the phytopathogens *Fusarium culmorum* and *F. oxysporum*. Furthermore, even the lowest concentrations of fucosterol (0.05% and 0.2%) were observed to degrade the structure and lyse the macroconidia of phytopathogens to a greater extent [85]. Besides, fucosterol from *S. linearifolium* brown seaweed) displayed antiplasmodial activity against the malarial parasite 3D7 chloroquine-sensitive *P. falciparum* by intervening at the schizont stage. Compared with chloroquine (IC_50_ of 12.81 μg/mL), fucosterol (IC_50_ of 7.48 μg/mL) presented higher plasmodial activity [86]. Similarly, assessment of antimalarial activity against the FCM29 strain of *P. falciparum* using the crude extract of *S. incisifolium* significantly inhibited the growth of the organism, with an IC_50_ value of 57.80 ± 1.91 μg/mL [81]. Overall, these findings underscore the antimicrobial properties of seaweed-based phytosterols. Generally, sterols target the cell walls of the microbes. It blocks the biosynthesis of the cell wall and makes the cells more susceptible to osmotic lysis [87,88]. However, the mechanism of action remains poorly studied in seaweeds-derived phytosterols.

### 4.3. Phytosterols as Anti-Inflammatory Agents

Chronic inflammation generally develops as part of the sequence of cellular events following acute inflammation in humans and animals. The important microcirculatory responses, including the recruitment of leukocytes, vascular permeability modulation, and releasing inflammatory mediators, are responsible for developing and maintaining inflammation [89,90]. The inflammatory cytokines, including tumor necrosis factor-α (TNF-α), cyclooxygenase (COX-2), interferon gamma (IFNγ), interleukin (IL)-1β, IL-6, IL-8, and IL-10, are the key modulators of low-grade chronic inflammation [91,92]. Lipopolysaccharide (LPS) triggers an immune response by interacting with the membrane receptor to enhance the production of cytokines. The excessive production of such inflammatory mediators contributes to chronic inflammatory disorders. Chronic inflammation has been subjected to the physiopathology of several diseases, including cancer, hepatitis, obesity, diabetes, metabolic syndrome, arthritis, degenerative neurological disorders, and cardiovascular diseases [93]. Several bioactive natural compounds have shown anti-inflammatory activity to protect against chronic diseases. Recently, numerous anti-inflammatory agents have been isolated from marine algae with protective efficacy against chronic inflammation [94,95]. In particular, the mechanisms involved in counteracting neuroinflammation include inhibiting the expression of pro-inflammatory enzymes [96], reducing inflammatory mediators, modulating MAPK pathways [97], and NK-κB activation [98]. Among the marine algae derivatives, phytosterols have been described as efficient anti-inflammatory agents. Among chronic inflammations, inflammatory bowel diseases (IBDs) cause serious problems in younger generations, as they cause serious illnesses and are proven to be fatal, if untreated [99]. In the hunt for natural compounds to treat such inflammations, phytosterols had been proven to augment the clinical remission of IBDs in mice [100]. However, there is no report that can link these effects to macroalgae-derived phytosterols.

Fucosterol and its derivatives are the most common phytosterols, majorly found in brown seaweed [7]. Several studies have investigated the anti-inflammatory potential of fucosterol and its derivatives. For instance, fucosterol, isolated from the brown seaweed *E. bicyclis*, has been shown to attenuate the expression of inflammatory mediators such as inducible nitric oxide (NO) synthase and COX-2 in LPS-stimulated macrophages [66]. Sun et al. [101] also reported that fucosterol shows anti-inflammatory activity by suppressing TNF-α, IL-6, and IL-1β expressions. Fucosterol also attenuates the LPS-induced inflammatory response by suppressing the production of TNF-α, IL-6, and IL-1β and nuclear factor-κB (NF-κB) activation in LPS-induced alveolar macrophages [102]. NF-κB has been considered a prototypical pro-inflammatory signaling pathway. The activation of the NF-κB, by nuclear translocation of cytoplasmic complexes, plays a key role in inflammation via the induced transcription of genes encoding pro-inflammatory mediators [103]. Also, the anti-inflammatory agent fucosterol has been isolated from the most abundant marine brown algae, *S. binderi.* Fernando et al. [71] have reported that the fucosterol from *S. binderi* inhibited chronic inflammatory responses by suppressing the production levels of TNF-α and IL-6, COX2, and prostaglandin E2, the nuclear translocation of both p65 and p50, and the activation of the p38 mitogen-activated protein kinase (MAPK) pathway [71]. Alzheimer’s disease, inflammatory bowel disease, and rheumatoid arthritis are postulated to be synchronized, in part, by MAPK p38 [104,105,106]. The activated p38 pathway triggers the production of pro-inflammatory mediators (IL-1β, TNF-α, IL-6, and COX-2) that direct the remodeling of connective tissue in pathological conditions by encoding adherent proteins along with other inflammatory-related molecules [107]. Another study found that fucosterol, isolated from *U. pinnatifida*, reduced the formation of pro-inflammatory cytokines (iNOS, TNF-α, and IL-6) and, subsequently, inhibited LPS-stimulated NO production by suppressing the activation of NF-κB and the phosphorylation of p38 MAPK in macrophages [108].

Phytosterols present in seaweed have been shown to be neuromodulators in the central nervous system (CNS). By stimulating synapse remodeling, improving neuroinflammation, and suppressing neurotoxic protein accumulation in the CNS, seaweed-derived phytosterols can be considered an exciting therapy for the treatment of neurodegenerative diseases [13]. Alzheimer’s disease is attributed to the accumulation of the toxic protein amyloid-β (Aβ) in the CNS. Although Aβ is crucial for neuronal survival and synaptic plasticity, high concentrations eventually lead to cell death and neurotoxicity [109]. Wong and colleagues reported that fucosterol also protects against Aβ-associated neuroinflammation via attenuating the production of inflammatory mediators in Aβ-induced microglial cells [68]. Aβ peptide was found to trigger pro-inflammation through the activation of p38 MAPK [110]. Chronic microglial activation by Aβ stimulated the expression of inflammatory mediators such as TNF-α, IL-1β, IL-6, NO, and prostaglandin E2, which could lead to the neuroinflammation known as Alzheimer’s disease [111,112]. Also, the anti-inflammatory agent fucosterol has been shown to be a non-competitive inhibitor of the enzyme β-secretase, which is important for the production of toxic Aβ monomers [113]. 

Saringosterol, another important seaweed-derived phytosterol, has been found to diminish the formation of neuronal Aβ while triggering microglia-mediated Aβ clearance [114]. The brown seaweed *S. fusiforme* contains high ranges of 24(S)-saringosterol. Saringosterol reduced the load of Aβ plaque and improved cognitive performance in an AD animal model [113]. However, with the exception of saringosterol, seaweed-derived fucosterol, fucoxanthin, and fucoidan have been reported to act against memory deficits [115]. Overall, these findings highlight the anti-inflammatory properties of seaweed-derived phytosterols.

### 4.4. Phytosterols as Anticancer Agents

**Figure 5:**
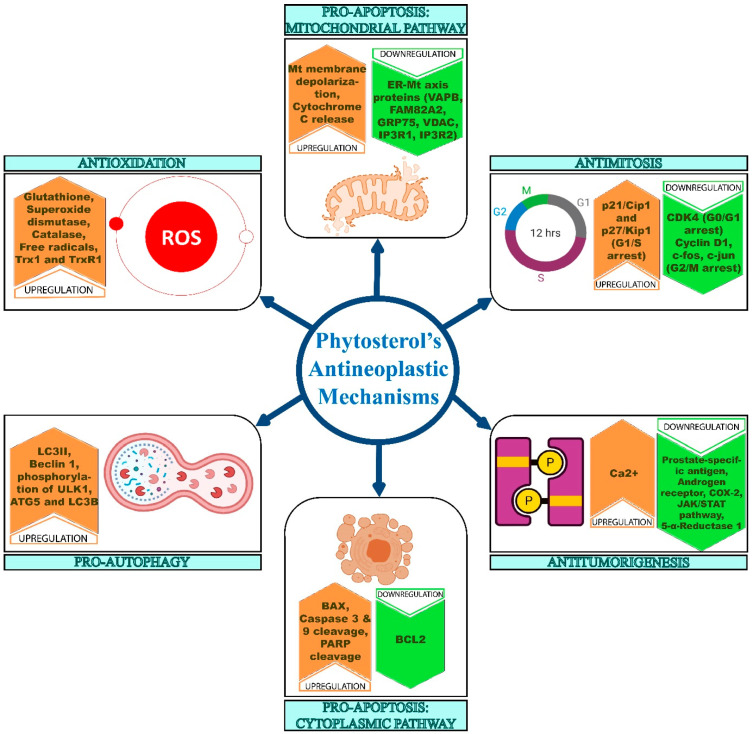
Anticancer mechanisms of phytosterols. The different modes of action giving rise to the anticancer properties of phytosterols act in different gene pathway. Controlled regulation of genes involved in antioxidation, apoptosis and antimitosis restricts the growth of the cancer cells.

Foods enriched with phytosterols and their oxy- derivatives could help to prevent tumor growth in humans. Phytosterols play various roles in the human body to control the progression of the numerous cancer types [116]. In gastric cancer cells (SNU-1), stigmasterol induces mitochondrial-mediated apoptosis, through which it suppresses cancer development. The activation of the apoptotic pathway by stigmasterol was evidenced by increased Bax and decreased Bcl-2 expression. In a concentration-dependent manner, it also reduced cancer cell migration and caused G2/M cell cycle arrest. In addition, stigmasterol suppresses the JAK/STAT signaling pathway, which is thought to be responsible for its anticancer effect in SNU-1 cells [117]. In Swiss albino mice, the effect of stigmasterol on 7,12-dimethylbenz[a]anthracene (DMBA)-induced skin cancer was examined. In the stigmasterol-treated groups, the tumor size and the total number of papillomas were decreased. Meanwhile, GSH, SOD, and catalase activity were all elevated in the skin of stigmasterol-treated mice. Furthermore, stigmasterol treatment dramatically reduced high levels of lipid peroxide and DNA damage in the control group. This implied that stigmasterol protected against DMBA-induced genotoxicity by acting as an antigenotoxic and antioxidant [118]. In a time- and dose-dependent way, stigmasterol dramatically reduced the viability of gastric cancer cell lines, such as SGC-7901 and MGC-803. Changes in the expression of the apoptotic key proteins Bax and Bcl-2, the cleavage of caspase-3, and poly (ADP-ribose) polymerase (PARP) have indicated apoptotic-mediated cell death upon treatment with stigmasterol in both cell lines [119]. LC3-II acts as a marker for an autophagic activity [120]. Treatment with stigmasterol resulted in an increase in LC3-II levels, indicating the activation of autophagy. Similarly, beclin 1 is an upstream molecule that is essential for autophagosome formation and that plays a key role in autophagy induction [121]. The expression of beclin-1 was also enhanced upon stigmasterol treatment, which further confirmed autophagy activation (Figure 5). As a result, stigmasterol’s anticancer impact in gastric cancer cells is dependent on apoptotic and autophagy-mediated mechanisms [119]. The overall mechanisms of the anticancer activities of phytosterols have been illustrated in Figure 5.

Campesterol inhibited the aggregation of the human ovarian cancer cell lines ES2 and OV90. In both cell lines, it increased the expression of proapoptotic proteins, promoted caspase 3 and caspase 9 cleavage, and increased the expression of cytochrome C, BAK, and BAX. Campesterol also elevates the levels of autophagy-related proteins like BECN1, phosphorylated (p)-ULK1, ATG5, and LC3B (Figure 5). The amount of calcium in ES2 cells was observed to be higher after treatment with campesterol. In addition, campesterol exposure elevates the expression of the ER–mitochondria axis proteins, such as VAPB, FAM82A2, GRP75, VDAC, IP3R1, and IP3R2 [122]. The new formation of capillary blood vessels is called angiogenesis. It is considered as major hallmark of cancer development and metastasis. The major steps in angiogenesis include the proliferation of endothelial cells, differentiation, migration, the destruction of the extracellular matrix, tube-like structure formation, and the formation of vessel branches [123]. Campesterol has been shown to reduce bFGF-induced pathological angiogenesis in HUVEC endothelial cells. The CAM assay confirmed that campesterol significantly hindered the creation of new embryonic blood vessels without impacting the established vasculature [124].

Brassicasterol treatment has been found to reduce the viability of LNCaP and PC-3 cells. It reduced the number of LNcaP cells and suppressed the expression of prostate-specific antigen and androgen receptors. Also, brassicasterol caused sub-G1 phase arrest. The expression of PARP was inhibited by brassicasterol treatment, during which induced cleaved caspase-3 expression was noted. Brassicasterol inhibits cell migration in LNCaP cells, as well [125]. Furthermore, it inhibited n-butyl-(4-hydroxybutyl) nitrosamine (BHBN) and SS-induced carcinogenesis. Cyclin D1 has been found to be overexpressed in bladder epithelial cells and it is a key player in the cell cycle [126]. When compared to the carcinogenesis group, the expression levels of cyclin D1, c-fos, and c-jun were significantly lower in brassicasterol-treated mice. This indicates the occurrence of cell cycle arrest. Prostaglandin E2 (PGE2), which is produced by the enzyme cyclooxygenase-2 (COX-2), also plays a role in the development of bladder cancer [127]. Brassicasterol treatment significantly reduced the expression of COX-2 in rats with comparison to the cancer-induced rats. Testosterone has a key role in bladder cancer development, while 5-α-reductase converts testosterone to the active metabolite dihydrotestosterone (DHT). DHT has androgenic properties because it interacts with the nuclear androgen receptor (AR). When rats treated with brassicasterol were compared to cancer-induced rats, the expression level of 5-α-R1 was considerably lower. Similarly, treatment with brassicasterol also dramatically reduced the expression levels of 5-α-reductase type 2 (5-α-R2) and AR, compared with the cancer-induced rats. According to these findings, brassicasterol inhibits bladder carcinogenesis by acting on cell cycle-associated signaling and androgen signaling via several mechanisms [128].

β-Sitosterol treatment caused DNA fragmentation and tailing in the COLO 320 DM human colon cancer cell line. In COLO 320 DM cells, it also resulted in the production of free radicals. In vitro, β-sitosterol treatment dramatically reduced β-catenin and PCNA expression in COLO 320 DM cells. Similarly, in vivo, β-sitosterol treatment for 16 weeks significantly reduced the number of aberrant crypts and crypt multiplicity in DMH-treated rats [129]. At 48 h, β-sitosterol significantly inhibited cancer cell proliferation in MDA-MB-231 cells. It triggered G0/G1 arrest, as shown by lower levels of CDK4 and cyclin D1 proteins and higher levels of p21/Cip1 and p27/Kip1 proteins. Apoptotic activation was also confirmed by Bcl-2 downregulation and Bax upregulation. The depolarization of mitochondrial membrane potentials is caused by β-sitosterol-induced apoptosis [130]. The cytotoxic effects of seaweed-derived compounds pertaining to anticancer activity are listed in Table 2. 

The effects of β-sitosterol on A549 cells was significant, with concentration- and time-dependent action. β-sitosterol-treated Swiss albino mice were healthy and showed no significant changes in behavior or mortality rate. A549 cells treated with β-sitosterol showed nuclear and DNA fragmentation with apoptotic morphological changes. β-sitosterol treatment caused the decreased expression of Bcl-2 and upregulated the expression of Bax protein; it also resulted in the cleavage of caspase-3 and PARP protein. β-sitosterol induced mitochondrial membrane depolarization and released cytochrome c, which confirmed that the mode of apoptosis induced by β-sitosterol in A549 cells was intrinsic. Furthermore, when cells were treated with β-sitosterol, the number of cells in the G2/M phase was increased [131]. The effect of β-sitosterol in A549 cells was mediated by elevated ROS, which was confirmed by the pretreatment of NAC. In cancer cells elevated levels of thioredoxin (Trx1) and thioredoxin reductase (TrxR1) play a role in ROS homeostasis [132]. β-sitosterol decreased the expression of both TrxR1 and Trx1 in A549 cells thereby causing oxidative stress and intrinsic mode of cancer cell death [133].

## 5. Safety of Phytosterols

Still today, clinical safety trials using both animal and plant models have not reported any negative effects of phytosterols [4]. Even though previous studies have specifically focused on the negative influence of phytosterol on sex hormones and organs, recent safety evaluations showed that phytosterol did not have an estrogenic impact on a rabbit animal model [154]. Subsequent studies also revealed that the daily administration of phytosterol (8.6 g) for 3 weeks to humans seems have no negative effect on gut microbiota and sex hormones [155]. Furthermore, it also demonstrated that the consumption of phytosterol at a very low dose does not have a serious effect on reproductive organs. However, some studies have demonstrated adverse clinical events related to plant sterols’ consumption [156]. It has been reported that consumption of phytosterol-enriched foods (olive oil and canola and soybean oils) decreases the life span of stroke-prone spontaneously hypertensive rats [157] but there is no report on seaweed-derived phytosterols. Other potential risks related with the intake of phytosterol is occurrence of rare inherited disorder phytosterolemia [158]. However, a myriad of research studies on phytosterol have inevitably proved that intake of phytosterol has potential health benefits, hence it is suggested that phytosterols-rich foods such as seaweed can be used as functional foods [159]. Phytosterols are sensitive to oxidation-like cholesterol, resulting in the formation of phytosterol oxidation products by enzymatic or non-enzymatic mechanisms during heat processing, storage, and human metabolism [160,161]. Despite the low concentration of cholesterol oxidation products in food, they have been demonstrated to cause cytotoxicity and genotoxicity. Therefore, the safety of phytosterol oxidation products has been studied and found to have cytotoxic effects on different cell lines [162].

## 6. Conclusions and Future Perspectives

Drug resistance, a natural phenomenon through which pathogenic microbes develop resistance against therapeutic agents, has become a major threat to public health. The development of resistance to conventionally available drugs has led to a search for novel therapeutic alternatives. At present, several synthetic drugs are being used, however, they often cause toxic side effects and become ineffective when used for a long time. Therefore, the search for new therapeutic agents from natural sources is expected to yield suitable alternatives. This review illustrated the implication of seaweed-derived phytosterols with their biosynthetic pathways and mechanisms of action in an effort to help in understanding their significant biomedical applications and valuable health benefits. Additionally, future drug designs, through the utilization of the structure determination and pharmacokinetics of seaweed sterols, will pave the way for developing novel therapeutic applications. The presence of significant amounts of steroids in seaweeds, especially cholesterol, campesterol, fucosterol, β-sitosterol, and stigmasterol, could contribute to several beneficial biological activities. Bioactive sterols as drugs could mediate multi-attribute target-oriented pharmacological activity, such as anticancer, antimicrobial, anti-inflammatory, and antioxidant activities. Consequently, phytosterols from seaweed were explored in order to establish the effectiveness of steroidal drug molecules in preventing drug resistance and reducing the side effects of pharmacological therapies. It is suggested that future research should focus on improving the bioavailability of phytosterols using various physical and chemical modifications. The preparation of phytosterol derivatives by chemical synthesis, such as enzyme-catalyzed synthesis and ionic liquid-catalyzed synthesis, is expected to increase their fat solubility. Physical changes, including the encapsulation of phytosterols in nano-delivery systems, can boost oral bioavailability by increasing solubility in the gastrointestinal tract, increasing absorption in targeted tissues, and ensuring sustained release. These can help us to improve the widespread use of these important natural bioactive compounds from seaweed in biomedical applications.

## Figures and Tables

**Table 1:** Examples of phytosterols identified from seaweeds.

Source	Extraction Method	Methods of Analysis	Identified Phytosterols	References
*Gelidium spinosum*	Soxhlet method	FTIR and GC–MS	Stigmasterol	[32]
*Saccharina latissima*	saponified extract	GC–MS	cholesterol, desmosterol, 24-methylenecholesterol, fucosterol, cycloartenol, and unknown ∆^5^-sterol	[35]
*Palmaria decipiens*,* Plocamium cartilagineum*,* Iridaea cordata*, and* Pyropia endiviifolia*	alkaline hydrolysis	GC–MS	cholesterol, brassicasterol, campesterol, stigmasterol, β-sitosterol, and fucosterol	[33]
*Ecklonia radiata*	alkaline saponification	LC-MS/MS and GC–MS	fucosterol, Sitostanol, 24α-methyl cholesterol, and 24α-ethyl cholesterol	[36]
*Padina australis* and *Stoechospermum marginatum*, and *Ahnfeltiopsis pygmaea*	acid and alkaline hydrolysis followed by solvent extraction, derivatization, and GC determination	gas chromatography coupled with a flame ionization detection system (GC–FID)	sitostanol, campestanol ergosterol, campesterol, delta-5-avenasterol, stigmasterol, sistenol, cholesterol, and 24-methylenecholesterol	[37]
*Adenocystis utricularis*, *Desmarestia confervoides*, *Curdiea racovitzae*, *Myriogramme manginii*, and *Ulva intestinalis*	Soxhlet method	GC–MS and FT-IR	fucosterol, cholesterol, and hydroxymethylcholesterol	[38]
Phaeophyta (*Cystoseira barbata*, *Cystoseira compressa*, *Fucus virsoides*) and chlorophyta (*Codium bursa)*	agitation-assisted extraction and pressurized liquid extraction	TLC	cholesterol, brassicasterol, campesterol, campestanol, stigmasterol, β-sitosterol, fucosterol, and isofucosterol	[39]
*Cystoseira trinodis*	solvent extraction and column chromatography	^1^H, ^13^C NMR, heteronuclear multiple-bond correlation (HMBC), heteronuclear single-quantum coherence (HSQC), GC–MS, and electron ionization-mass spectra (EI-MS)	saringosterol, β-sitosterol	[40]
*Sargassum horneri*	high-speed countercurrent chromatography	NMR	fucosterol and saringosterol	[41]
*S. fusiforme*	Folch method	GC–MS	24(S)-Saringosterol	[42]
*Halimeda tuna*,* Codium bursa*,* *and* Cystoseira barbata*	solvent extraction	GC and GC–MS	fucosterol, campesterol and β-sitosterol	[43]
*Sargassum elegans*	solvent extraction and column chromatography	NMR (^1^H and ^13^C), IR and mass spectral data	β-sitosterol, fucosterol	[44]
Phaeophyta (*Cystophora pectinata*, *Pyllospora comoasa*, *Scytothalia dorycarpa*, *Carpoglossum confluens*, *E. radiata*, *Sargassum lacerifolium*, *Perithalia caudata*, *Codium harveyi*, *Scytothalia dorycarpa*, *Hypnea valida*, *Cystophora monilifera*, *Hormosira banksia*, *Myriodesma integrifolium*, Epiphytic algae sp., *Cystophora subfarcinata*), Rhodophyta (*Austrophyllis harveyana*, *Rhodophyllis membaneacea*), and chlorophyta *(Codium fragile)*	maceration	post-chromatographic derivatization and HPTLC	β-sitosterol	[45]
*Ascoseira mirabilis*, *A. utricularis*, *Desmarestia anceps*, and *Phaeurus antarcticus*	saponification	GC–MS	cholesterol, desmosterol, brassicasterol, campesterol, stigmasterol, fucosterol, and β-sitosterol	[46]
*Ecklonia stolonifera*	silica gel column chromatography	^1^H and ^13^C NMR	fucosterol	[47]
Rhodophyta (*Gracilaria vermiculophylla*, *Pterocladiella tenuis*, *Palisada intermedia*, *Chrysymenia wrightii*, *Gracilaria elegans*, *Grateloupia asiatica*, *Laurencia okamurae*) and Phaeophyta (*Eckloniopsi radicosa*, *Sargassum thunbergia*, *Ecklonia kurome*, *Eisenia arborea*, *Sargassum piluliferum*, *S. fusiforme*, *U. pinnatifida*, *Ecklonia cava*)	saponification	HPLC with fluorescence detection	cholesterol, β-sitosterol, ergosterol, stigmasterol, and fucosterol	[48]
*S. horneri*	total lipid extraction using methanol	RP-HPLC	fucosterol	[49]
*Hizikia fusiformein*	ethanol extraction and chromatographic separation	LC/ electrospray ionization (ESI)-MS	fucosterol	[50]
*A. utricularis*, *Ascoseira mirabilis*, *Cystosphaera jacquinotii*, *D. anceps*, *Durvillaea antarctica*, and *Himantothallus grandifolius*	ultrasound irradiation	LC-MS/MS	ergosterol, brassicasterol, fucosterol, β-sitosterol, campesterol, cholesterol, and stigmasterol)	[6]
*Porphyra dentata*	methanol extraction and silica gel column chromatography	HPLC- evaporative light scattering detector (ELSD)	cholesterol, β -sitosterol, and campesterol	[51]

**Table 2:** Cytotoxicity of seaweed-derived compounds toward anticancer activity.

Seaweed Names	Cell Lines Used	Therapeutic Compounds	Anticancer Activity	References
*Ulva fasciata*, *Ulva lactuca*,* Amphiroa anceps*,* Corallina mediterranea*, and* Sargassum filipendula*	human breast adenocarcinoma cell line (MCF-7) and colorectal carcinoma cell line (HCT-116)	palmitic acid, oleic acid, retinoic acid, dihydroactinidiolide, thiosemicarbazide, diisobutyl phthalate, and phytol	anticancer agents against human breast and colon cancers	[134]
*Sargassum* spp.	SMMC-7721, Huh7, and HCCLM3 liver	fucoidan	deactivates the integrin αVβ3/SRC/E2F1 signaling pathway; Antimetastatic	[135]
*Ulva lactuca*,* Codium tomentosum*,* Cystoseira crinita*,* Cystoseira stricta*,* Sargassum vulgare*,* Gelidium latifolium*,* Hypnea musciformis* and *Jania rubens*	human colorectal carcinoma (Caco2) and human corneal epithelial cells (HCEC)	polyphenols and flavonoids	human colorectal carcinoma	[136]
*Ecklonia maxima*	HeLa, H157 and MCF7 cancer cell lines	phlorotannins and sterol	cytotoxic activity	[137]
*Sargassum* spp., *Turbinaria* spp. and *Padina* spp.	breast cancer (MCF-7) and colon cancer cells (WiDr)	fucoidan	showed potential selective cytotoxicity	[138]
*Sargassum hemiphyllum*	*HCT116*	oligo-fucoidan	DNA damage; cell cycle checkpoint; prevents HCT116 tumorigenicity and regulate the cancer cell death	[139]
*Ulva lactuca* and *Eucheuma cottonii*	breast MCF-7 and colorectal HCT-116 cancer cells	steroids, glycosides, flavonoids, and tannins	anti-breast and anticolorectal cancer agents	[140]
*Carpodesmia tamariscifolia*	hepatocellular carcinoma Hep G2, AGS and HCT-15 cell lines	isololiolide	caspase-3 activation, decreased Bcl-2 levels, increased p53 expression and PARP cleavage	[141]
*Fucus vesiculosus*	human hepatoma cell line MHCC-97H	fucoidan	macrophages M2 anti-inflammatory reduction; inhibition of tumor cell migration	[142]
*Brown algae* spp.	MCF-7 cell line	phloroglucinol	decreased CD44+ cancer cell population, expression of CSC regulators such as Sox2, CD44, Oct4, Notch2, and β-catenin; inhibited KRAS and its downstream PI3K/AKT and RAF-1/ERK signaling pathway	[143]
*U. pinnatifida*	human hepatocellular carcinoma SMMC-7721 cells	fucoidan	apoptosis via the ROS mediated mitochondrial pathway	[144]
*U. pinnatifida*	PC-3 human prostate cancer cells	fucoidan	induced intrinsic and extrinsic apoptosis pathways	[145]
*Laminaria* *digitata*	HT-29 colon cancer cells	laminarin	induction of apoptosis; affected insulin-like growth factor (IGF-IR); decreased MAPK and ERK phosphorylation; decreased IGF-IR-dependent proliferation	[146,147]
*Sargassum muticum*	MCF-7 cells	*Sargassum muticum* methanol extract (SMME)	induced apoptosis; showed antiangiogenic activity in the chorioallantoic membrane (CAM) assay; antioxidant effects	[148]
*Porphyra dentata*	4T1 cancer cells	cholesterol, β-sitosterol, and campesterol	induced apoptosis; decreased the ROS and arginase activity of MDSCs in tumor-bearing mice	[51]
*Sargassum* spp.	MCF-7 (breast cancer) and Hep-2 (liver cancer) cell line	ethanol extract	induced cell shrinkage, cell membrane blebbing and formation of apoptotic bodies	[149]
*U. pinnatifida*	A549 human lung carcinoma cells	fucoidan	induced apoptosis through down-regulation of p38, PI3K/Akt, and the activation of the ERK1/2 MAPK pathway	[150]
*Sargassum oligocystum*	K562 and Daudi human cancer cell lines	fucoidans	antitumor activity	[151]
*U. pinnatifidasporophylls*	leukemia A20 cells	fucoidan	T-cell mediated and NK cell response; tumor destruction by immune cells	[152]
*Amphiroa zonata*	human leukemic cells	palmitic acid	showed selective cytotoxicity	[153]
